# Cosine Similarity Distillation Vision Mixture-of-Experts for Intelligent Housing-Dimensional Urban Physical Examinations

**DOI:** 10.3390/s26113473

**Published:** 2026-05-31

**Authors:** Kun Zhao, Helei Ren, Wenbin He, Yuhong Zhao, Jinming Jiang, Wanxiang Yao, Weijun Gao, Qichao Ban

**Affiliations:** 1 School of Information Management, Qingdao University of Technology, Qingdao 266520, China; sterling1982@163.com (K.Z.); heleiren2026@163.com (H.R.); binbin12019@outlook.com (W.H.); 2 College of Architecture and Urban Planning, Qingdao University of Technology, Qingdao 266520, China; yuhongzhao9201@163.com; 3 Innovation Institute for Sustainable Maritime Architecture Research and Technology (iSMART), Qingdao University of Technology, Qingdao 266033, China; jiangjinming@qut.edu.cn (J.J.); yaowanxiang@qut.edu.cn (W.Y.); gaoweijun@me.com (W.G.); 4 Faculty of Environmental Engineering, University of Kitakyushu, Kitakyushu 802-8577, Japan

**Keywords:** old residential, intelligent urban physical examination, mixture of experts, image classification, complex visual scenes

## Abstract

Intelligent housing-dimensional urban physical examination requires evaluating complex visual scenes in aging communities. Existing methods and datasets are insufficient for these heterogeneous tasks and severe class imbalances. To address this, we introduce the **H**ousing-dimensi**O**nal vis**U**al in**S**pection imag**E D**ataset (**HOUSED**) with a hierarchical labeling scheme, and propose a hierarchical Vision Mixture of Experts (VMoE) framework. At its core, the proposed CS-DisVMoE module utilizes a CS-Soft routing mechanism to capture spatial feature correlations, optimizing expert assignment and reducing inference overhead. Additionally, a FENNEL-based non-linear graph partitioning mechanism converts pre-trained dense weights into semantically coherent expert initializations, accelerating convergence while preserving localized visual clustering. To address the hierarchical labels, we design a composite loss function: a Supervised Contrastive Loss acts as a parent-category soft constraint to accelerate convergence, while Focal Loss mitigates data imbalance and handles fine-grained subcategory classification via hard sample mining. Across evaluated datasets, the full proposed framework improves accuracy by an average of 4.3% over the ViT-Tiny baseline and 1.81% over the best-performing VMoE baseline. Furthermore, it achieves these improvements with lower computational costs. Further tests on mixed public vision datasets verify its generalizability and competitive performance for complex-scene applications.

## 1. Introduction

Aging residential communities often suffer from prevalent structural and functional issues, including deterioration of buildings, inadequate facilities, and unauthorized construction. These deficiencies not only undermine the safety, functionality, and comfort of living in buildings, but also pose significant safety hazards for residents [1,2,3]. Consequently, in the context of urban renewal, renovating existing buildings represents a more resource-efficient alternative to demolition and reconstruction [4]. Therefore, inspecting structural damage and unauthorized use of facilities in aging residential communities has become a critical component of the housing-dimensional urban physical examination.

The rapid development of computer vision technology has provided efficient tools for automated urban physical examinations [5,6]. In 2017, Yang et al. [7] constructed the Concrete Structure Spalling and Crack (CSSC) database for building defect detection by collecting real-world concrete crack images in Manhattan and crawling relevant online data. By fine-tuning the VGG16 model on the CSSC dataset, they achieved a detection accuracy exceeding 93%. More recently, in 2024, Kottari et al. [8] constructed the Building Defects Detection Dataset (BD3) covering six types of building wall defects (algae, large cracks, small cracks, spalling, peeling, and stains) and conducted benchmark evaluations across five baseline models.

**Figure 1:**
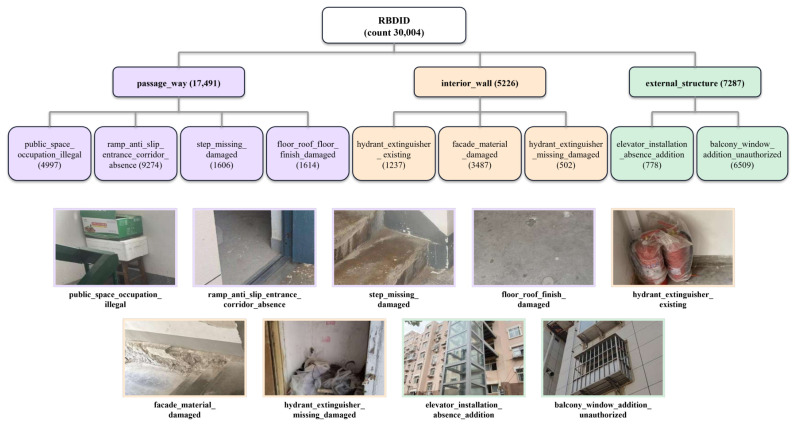
Hierarchical label taxonomy and representative visual samples of the HOUSED dataset. Colors distinguish the three parent categories, and the numbers denote the corresponding sample counts.

However, existing studies primarily focus on detecting specific defects (e.g., concrete cracks). In contrast, comprehensive urban physical examinations in aging residential communities involve highly complex visual scenes and significantly diverse sub-tasks. As illustrated in Figure 1, these deficiencies span multiple spatial contexts, including public passageways, interior walls, and external structures. Furthermore, each specific issue typically exhibits complex and highly heterogeneous visual features.

Based on the city physical examination guidelines issued by the Ministry of Housing and Urban-Rural Development of China and relevant national standards [9,10,11], we introduce the concept of housing-dimensional urban physical examination, which refers to the comprehensive visual inspection of aging residential communities across multiple spatial contexts (e.g., passageways, interior walls, and external structures) to assess structural and functional deficiencies. This poses a significant challenge to traditional detection models. Furthermore, there remains a lack of comprehensive datasets tailored for housing-level urban physical examinations. To address this, our research team captured extensive on-site imagery of various structural and functional deficiencies across four aging residential communities in Qingdao. We then hierarchically annotated these data based on their specific spatial contexts (see Section 3), thereby constructing the **H**ousing-dimensi**lO**nal vis**U**al in**S**pection imag**E D**ataset **(HOUSED)**.

In recent years, the Mixture of Experts (MoE) architecture has demonstrated exceptional performance in natural language processing and computer vision through multi-expert modules and expert routing mechanisms, particularly excelling in handling complex tasks across diverse scenarios [12]. Traditional sparse routing mechanisms forcibly assign specific tokens to a limited number of experts [13]. This approach performs well in natural language processing, where the data abstraction level is high and tasks (e.g., mathematical reasoning or translation) are relatively independent. However, in visual domains, there is often an underlying perceptual consistency across different scenes. This “hard assignment” strategy ignores the correlations among multiple scenes, often resulting in suboptimal performance in vision tasks. Conversely, the soft routing mechanism calculates weights for all visual tokens across all experts [14], which improves MoE performance in vision tasks to some extent but significantly increases computational costs. Moreover, its simple weighting mechanism often exhibits limited representational capacity when dealing with stronger consistency relationships beyond basic visual perception, such as the spatial geometric consistencies observed among different parent categories in the HOUSED dataset.

Therefore, we propose a Cosine Similarity Soft (CS-Soft) routing mechanism. This mechanism calculates the similarity between samples and leverages the generated soft routing to activate specific parts of the expert network within the MoE layer, thereby reducing computational overhead during inference while enhancing model performance. To address the issue of strongly correlated features being rigidly segmented in complex visual scenarios, and to further reduce the number of model parameters, we introduce an expert distillation method based on FENNEL (non-linear streaming graph partitioning) [15]. Compared to other expert upcycling approach, FENNEL converts high-quality dense weights into semantically coherent expert initializations while preserving the localized clustering of visual features through a non-linear penalty mechanism optimized by modularity, further accelerating the model’s training convergence.

In summary, the proposed core module is termed CS-DisVMoE (Cosine Similarity-Distilled Vision Mixture of Experts). On this basis, considering that the parent categories in the HOUSED dataset consist of only three categories without an obvious long-tail distribution, and that the ultimate classification objective is the accuracy of the fine-grained subcategories, we discard the rigid classification penalty for the parent categories. Instead, we innovatively combine a Supervised Contrastive Loss (adapted from the InfoNCE Loss function [16]) and Focal Loss [17] to propose a novel composite hierarchical loss, $L_{par-sub}$. By treating samples within the same parent category as positive pairs and those from different parent categories as negative pairs, this supervised contrastive learning approach minimizes the distance between positive pairs and maximizes the variance between negative pairs in the feature space. This establishes a “soft” contextual foundation for the parent categories. Simultaneously, Focal Loss is applied to fine-tune subcategory classification, increasing the penalty distinction between high- and low-confidence prediction regions via a steeper loss curve to facilitate hard sample mining. This synchronously accomplishes both parent-category contextual assistance and fine-grained subcategory tuning within a single training session.

The main contributions of this paper are summarized as follows:

- **The HOUSED dataset is constructed.** We compiled and constructed the **H**ousing-dimensi**O**nal vis**U**al in**S**pection imag**E D**ataset **(HOUSED)** for housing-dimensional urban physical examinations. Addressing prevalent inspection issues, we defined a hierarchical semantic label system comprising 3 parent categories and 9 fine-grained subcategories.
- **The CS-Soft routing mechanism is proposed.** A cosine similarity metric is utilized to match MoE inputs with expert slot weights. Furthermore, the original data information is fully preserved through an inverse hyperbolic tangent transformation to better represent visual correlations across different spatial contexts.
- **A FENNEL-based expert distillation method is proposed.** In this study, expert distillation refers specifically to converting a pre-trained dense model into a MoE architecture through structurally informed weight initialization, rather than conventional knowledge distillation. A non-linear streaming graph partitioning method (FENNEL) is employed to reorganize pre-trained dense MLP weights into semantically coherent expert groups. This effectively reduces model training time and computational overhead while enhancing overall performance.
- **A composite hierarchical loss function ($L_{par-sub}$) is proposed.** By fully utilizing hierarchical labels, this loss synergistically integrates Supervised Contrastive Learning to accelerate convergence, and Focal Loss to mitigate subcategory data imbalance and facilitate hard sample mining, thereby significantly enhancing feature representation and fine-grained classification accuracy in complex scenes.

## 2. Related Work

### 2.1. Vision Mixture of Experts Model

Jacobs et al. [18] first proposed the concept of MoE in 1991. Shazeer et al. [19] introduced the sparsely-gated MoE in 2017, significantly advancing its development. Subsequently, MoE was applied to visual tasks [20]; however, most early research efforts concentrated on multi-scale feature learning and local part representation within convolutional neural networks. V-MoE [21] first utilized the Vision Transformer (ViT) as its backbone, introducing sparsely-gated MoE into computer vision to mitigate the high computational cost of dense models while improving scalability and efficiency.

Since 2024, most VMoE research has focused heavily on model optimization in multi-modal contexts. DeepSeek-VL2 [22] optimized hybrid-modal MoE through dynamic chunked visual encoding, addressing challenges in high-resolution processing, computational efficiency, and task diversity. MoNE [23] mitigated redundant calculations in ViT and parameter inflation in traditional MoE through a nested structure and dynamic routing. Yang et al. [24] focused on multi-modal VMoE training optimization, resolving gradient conflicts caused by different tokens assigned to the same expert. Despite these advancements, contemporary MoE models still possess massive parameter counts and demand significant computational resources.

### 2.2. Expert Routing Mechanism

The routing mechanism is a crucial factor distinguishing dense MoE from sparse MoE. Dense MoE models activate all experts during training, resulting in high computational costs. In contrast, sparse MoE models selectively activate specific experts during training and inference, achieving higher accuracy with fewer computational resources. In 2021, Lewis et al. [25] modeled token assignment as a linear assignment problem, ensuring each expert receives exactly the same number of tokens, which simplified the training process. Roller et al. [26] proposed splitting the linear layer of the expert module into multiple segments using hash functions, eliminating the need for load-balancing losses and complex assignment algorithms. Shazeer et al. [19] proposed a routing strategy that greedily selects the top-*k* experts for each token. In 2023, Google researchers introduced SoftMoE [14], which performs soft assignment by calculating the weighted averages of all tokens; however, its underlying computational process remains essentially dense.

Beyond routing design, another important challenge in MoE models is how to initialize expert parameters efficiently without training all experts from scratch. Recent studies have therefore explored dense-to-MoE conversion strategies, while broader studies on sparse neural networks also provide useful motivation for reorganizing dense weights into expert groups. These studies are reviewed in the following subsection.

### 2.3. Dense-to-MoE Weight Conversion

Dense-to-MoE conversion, also known as expert upcycling, has recently emerged as an efficient alternative to training MoE models from scratch. Sparse Upcycling [27] initializes MoE experts by duplicating dense MLP weights, thereby transferring pre-trained representations into sparse expert modules. From a broader perspective, the lottery ticket hypothesis [28] suggests that dense neural networks may contain reusable substructures, which provides a general motivation for reorganizing pre-trained dense weights into specialized expert groups. More recently, MoE Jetpack [29] groups neurons according to similarity before expert assignment to improve semantic coherence during dense-to-MoE conversion.

Building on this line of research, the proposed FENNEL-based expert distillation constructs a co-activation graph from the MLP neurons of a pre-trained dense ViT and applies non-linear streaming graph partitioning to initialize semantically coherent expert groups. Compared with direct duplication or similarity-based grouping, the proposed method further introduces a non-linear capacity penalty to balance expert assignment while preserving local visual feature aggregation. This design improves expert initialization and routing stability in VMoE architectures.

### 2.4. Hierarchical Label Classification

Hierarchical classification is prevalent in both computer vision and natural language processing. In text classification, hierarchical data structures are typically divided into tree structures and DAG (Directed Acyclic Graph) structures [30]. Feng et al. [31] proposed the C2AE-DAGLabel algorithm based on the DAG structure of gene ontology. In image classification, Zhang et al. [32] proposed a hierarchical multi-label representation learning framework that maintains the hierarchical relationships between categories. Wehrmann et al. [33] designed the Hierarchical Multi-label Classification Network (HMCN), applicable to both tree and DAG structures. Yan et al. [34] introduced the Hierarchical Deep Convolutional Neural Network (HD-CNN) for large-scale visual recognition.

However, existing hierarchical classification methods typically apply uniform, rigid loss functions (e.g., standard cross-entropy) across all taxonomic levels. In real-world urban physical examinations, the data architecture is inherently asymmetrical. Parent categories are generally coarse-grained with limited cardinality, where imposing strict rigid constraints yields diminishing returns and risks restricting the representational capacity of the feature space. Conversely, fine-grained subcategories present a dual challenge: they not only encompass a multitude of easily confused, hard-to-classify samples, but also frequently exhibit severe data imbalance. Consequently, designing an adaptive mechanism that can simultaneously accommodate relaxed contextual alignment at the parent level, while effectively addressing both class imbalance and hard-sample mining at the subcategory level, remains an unresolved challenge in hierarchical visual tasks.

## 3. Data Acquisition

To ensure the representativeness of the dataset, this study targeted aging residential infrastructure. We utilized a Geographic Information System (GIS) platform to systematically screen residential buildings constructed before the year 2000 within Qingdao urban area. These aging residential buildings often exhibit diverse structural vulnerabilities due to early design standards and prolonged material degradation. By sampling four representative aging communities, we ensured the selected environments encompassed various architectural styles and maintenance conditions.

Subsequently, a team of trained researchers conducted comprehensive on-site inspections aligned with urban physical examination standards. Investigators captured diverse visual scenes under varying weather and lighting conditions to maximize the dataset’s real-world robustness. Photography strictly focused on areas exhibiting potential safety hazards, structural deterioration, and unauthorized modifications, resulting in an initial collection of 42,365 raw images.

To construct a high-quality data set, the raw data was subjected to a strict cleaning process that involved the manual removal of blurred, overexposed, or irrelevant images, along with strict privacy anonymization. Ultimately, 30,004 high-fidelity images were retained. To minimize background noise, these valid samples were uniformly cropped and resized. This geometric normalization ensured that each image contained only a single, centrally located detection target, thereby enhancing the model’s focus during feature extraction.

The annotation process followed a rigorous two-round protocol. Three trained annotators, each with at least two years of experience in building inspection or urban planning research, participated in the labeling. In the first round, two annotators independently labeled each image. In the second round, a senior expert reviewed all disagreements and made the final decision. The initial inter-annotator agreement was 87.3% for parent categories and 82.1% for subcategories before adjudication, reaching 100% after expert review. To protect privacy, a pre-trained privacy detection model was applied to automatically blur faces, license plates, and other personally identifiable information. The dataset has been publicly released on the Science Data Bank (https://doi.org/10.57760/sciencedb.28941 (accessed on 26 May 2026)). The annotation pipeline follows standard practices in computer vision dataset construction, ensuring reproducibility and transparency.

Guided by the policy guideline and national standards [9,10,11], and considering the regional housing characteristics of Qingdao, the retained HOUSED samples were annotated and categorized into 9 fine-grained subcategories according to the primary housing deficiencies observed in the collected images. Recognizing the correlation between defects and architectural locations, we organized these subcategories into a hierarchical taxonomy of 3 parent categories defined by their spatial contexts (e.g., passageways, interior walls, and external structures). This hierarchical scheme provides a multi-level semantic structure for representation learning. The specific categories and sample quantities of the HOUSED dataset are detailed in Table 1. For each subcategory, the table reports its readable class name, original dataset label, sample size, and semantic meaning.

## 4. Methods and Models

**Figure 2:**
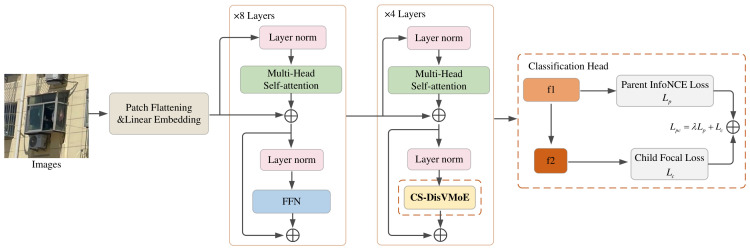
Overall architecture of the proposed model.

Figure 2 illustrates the overall architecture of the proposed model. Specifically, we adopt the ViT-Tiny [35] as the fundamental backbone, which comprises 12 sequential Transformer encoder layers. The data processing pipeline proceeds as follows:

First, to process the visual input, the original image undergoes patch embedding, flattening, linear mapping, and positional encoding to generate a sequence of input tokens. Subsequently, during the feature extraction phase, these tokens are processed sequentially by the 12 Transformer layers. To retain the robust baseline representation capabilities of the dense model while effectively managing computational overhead, the Multi-Layer Perceptrons (MLPs) within the first 8 layers remain unmodified as standard ViT blocks. Conversely, the MLPs in the remaining 4 deep layers are replaced by our proposed **CS-DisVMoE module** to handle complex feature routing. Finally, the extracted high-level features are fed into the classification head, where the entire network is jointly optimized using the improved composite hierarchical loss function, $L_{par-sub}$.

In our ViT-Tiny implementation, each input image is resized to 224×224 and divided into 196 patch tokens plus one class token, yielding m=197 tokens with an embedding dimension of d=192. The first 8 Transformer layers retain the original dense MLP blocks, while the last 4 layers are replaced with CS-DisVMoE modules. The number of experts is set to n=96 according to the ablation study, and each expert contains one slot (p=1), resulting in np=96 expert slots. The learnable projection matrices $W_{Q}$ and $W_{K}$ therefore have dimensions 192×192.

### 4.1. CS-Soft Routing Mechanism

CS-Soft follows the dispatch-and-combine paradigm of Soft MoE, while replacing dot-product routing logits with cosine-based token-slot affinities. For clarity, we describe the routing process for a single sample and omit the batch dimension. Let $T\in R^{m\times d}$ denote the input token sequence, where *m* is the number of tokens and *d* is the embedding dimension. In our ViT-Tiny implementation, m=197, including 196 patch tokens and 1 class token, and d=192. We use *n* experts and *p* slots per expert, resulting in np expert slots. In this study, n=96 and p=1.

The input tokens are projected into keys, and the learnable expert-slot embeddings are projected into queries:

(1) $$K=TW_{K},\;Q=E_{slot}W_{Q},$$

where $K\in R^{m\times d}$ denotes the projected token keys, $E_{slot}\in R^{np\times d}$ is a learnable expert-slot embedding matrix representing all np expert slots, $Q\in R^{np\times d}$ denotes the projected slot queries, and $W_{K},W_{Q}\in R^{d\times d}$ are learnable projection matrices.

The cosine affinity between expert slots and input tokens is computed as

(2) $$S=\mathrm{Norm}\left(Q\right)\mathrm{Norm}{\left(K\right)}^{⊤},\;S\in R^{np\times m},$$

where Norm(·) denotes row-wise $\ell_{2}$ normalization.

To enhance highly correlated token-slot pairs, we apply an inverse hyperbolic tangent transformation:

(3) $$U=\mathrm{arctanh}\left(S\right)=\frac{1}{2}\ln\frac{1+S}{1-S},\;U\in R^{np\times m}.$$

This transformation preserves low-similarity regions while enlarging differences among high-similarity pairs, thereby improving fine-grained token-slot discrimination. Compared with linear temperature scaling, arctanh adaptively amplifies high cosine-affinity regions as the similarity approaches 1, while keeping low-affinity regions relatively less affected. This provides a non-linear affinity enhancement that is difficult to reproduce with a single global temperature parameter.

For consistency with the Soft MoE formulation, we transpose U and define $A=U^{⊤}\in R^{m\times np}$, where each row corresponds to an input token and each column corresponds to an expert slot. The dispatch weights are computed by applying softmax over the token dimension:

(4) $${\tilde{D}}_{ij}=\frac{\exp(A_{ij})}{\sum_{i^{\prime }=1}^{m}\exp\left(A_{i^{\prime }j}\right)},\;\tilde{X}={\tilde{D}}^{⊤}T,$$

where $\tilde{D}\in R^{m\times np}$ and $\tilde{X}\in R^{np\times d}$. Each expert slot is therefore constructed as a convex combination of all input tokens.

The dispatched slot features $\tilde{X}\in R^{np\times d}$ are then fed into the corresponding expert networks. After expert processing, $Z\in R^{np\times d}$ denotes the expert outputs for all slots, where each row corresponds to one processed expert-slot feature. The combine weights are computed by applying softmax over the expert-slot dimension:

(5) $${\tilde{C}}_{ij}=\frac{\exp(A_{ij})}{\sum_{j^{\prime }=1}^{np}\exp\left(A_{ij^{\prime }}\right)},\;\tilde{Y}=\tilde{C}Z,$$

where $\tilde{C}\in R^{m\times np}$ and $\tilde{Y}\in R^{m\times d}$ denotes the reconstructed output token sequence. In this way, $\tilde{D}$ serves as the dispatch weight matrix, while $\tilde{C}$ serves as the combine weight matrix.

**Figure 3:**
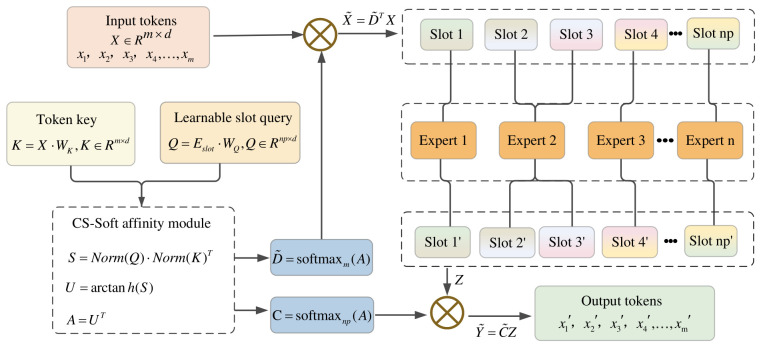
Schematic diagram of the Cosine Similarity Soft (CS-Soft) routing mechanism.

Thus, CS-Soft preserves the dispatch-and-combine formulation of Soft MoE, but introduces cosine similarity and arctanh-based affinity enhancement to achieve more discriminative routing for fine-grained recognition. In addition, the learnable expert-slot queries avoid dimensionally inconsistent reshaping of token features into expert slots, making the routing formulation consistent when m≠np. Figure 3 illustrates the detailed workflow of the CS-Soft routing mechanism.

### 4.2. FENNEL-Based Expert Distillation and Non-Linear Graph Partitioning

To reduce the training cost of MoE models and avoid training expert weights from scratch, we adopt a dense-to-MoE weight conversion strategy. The conversion pipeline consists of four stages: (1) pre-training a Dense ViT-Tiny on ImageNet-21k, (2) constructing a co-activation graph from the Dense model’s MLP layers, (3) partitioning the graph into expert groups using FENNEL, and (4) initializing the CS-Soft MoE layers from the partitioned weights. Specifically, the first 8 Transformer layers retain their original Dense weights, while the MLP layers in the last 4 layers (layers 9–12) are replaced with CS-Soft MoE layers initialized via FENNEL. In the housing-dimensional urban physical examination task, visual scenes exhibit high complexity and strong feature coupling. To overcome the limitations of linear penalty strategies, which tend to rigidly split semantically related features, we specifically introduce the FENNEL non-linear streaming graph partitioning algorithm.

The graph construction and partitioning procedure are detailed as follows. First, all training data are processed through a pre-trained ViT model, and the co-activation frequencies of neurons within the MLP layers are recorded to construct an undirected weighted co-activation graph G=(V,E). Here, *V* represents the graph vertices (neurons), and *E* represents the edges (co-activation frequency, indicating feature affinity). When traversing to a new neuron node *v*, the FENNEL algorithm determines its assigned expert partition $i^{*}$ by maximizing the following objective function:

(6) $$i^{*}=\arg\max_{i\in \{1,\ldots ,k\}}\left(|E\left(v,P_{i}\right)\left|-\alpha \gamma \right|P_{i}{|}^{\gamma -1}\right)$$

This objective function consists of two parts: the local aggregation term $\left|E(v,P_{i})\right|$, which encourages neurons with strong semantic associations to be routed to the same expert, and the non-linear capacity penalty term $\alpha \gamma |P_{i}{|}^{\gamma -1}$, which dynamically suppresses overloaded experts. The parameter γ controls the non-linearity of the penalty. A lower γ may provide insufficient capacity regularization and lead to expert congestion, whereas a higher γ may excessively penalize expert capacity and fragment semantically consistent visual features. The coefficient α is an adaptive scaling factor, calculated as $\alpha =\left|E\right|\frac{k^{\gamma -1}}{{\left|V\right|}^{\gamma }}$. The generated pseudocode is presented in Algorithm 1.
**Algorithm 1** FENNEL Expert Distillation Graph Partitioning Algorithm**Require:** Set of neuron nodes *V*, set of co-activation edges *E*, number of experts *k*, parameter γ=1.5**Ensure:** Expert index $i^{*}$ to which the neuron *v* is partitioned1:Calculate the scaling coefficient $\alpha =\left|E\right|\frac{k^{\gamma -1}}{{\left|V\right|}^{\gamma }}$2:**for** v∈V **do**3:   **for** i∈{1,…,k} **do**4:     Calculate local aggregation $E_{local}=\left|E\left(v,P_{i}\right)\right|$5:     Calculate non-linear penalty $Penalty=\alpha \gamma |P_{i}{|}^{\gamma -1}$6:     Calculate score $g\left(v,P_{i}\right)=E_{local}-Penalty$7:   **end for**8:   $i^{*}=\arg\max_{i}g\left(v,P_{i}\right)$9:   Add *v* to the set $P_{i^{*}}$10:**end for**11:**return** expert partition results

**Figure 4:**
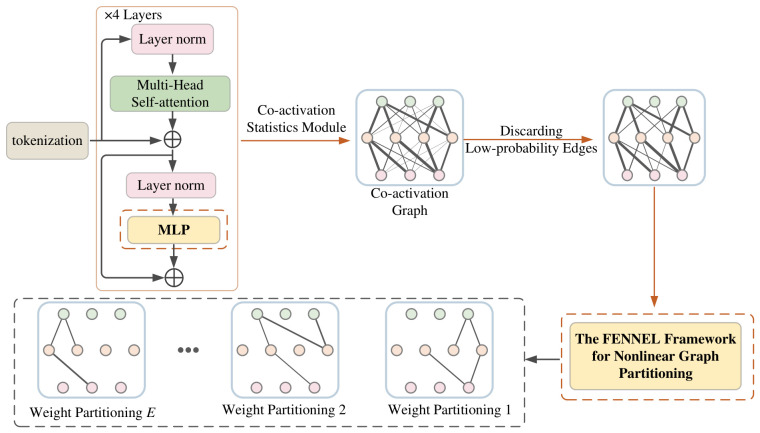
Illustration of the FENNEL-based expert distillation and non-linear graph partitioning. Different colors indicate different expert partitions formed during graph partitioning.

As demonstrated in Algorithm 1, the non-linear penalty value increases rapidly when the number of neurons $|P_{i}|$ in expert *i* approaches saturation. This ensures robust load balancing among experts and prevents the overloading of a single expert, while preserving local visual feature aggregation as much as possible. A schematic diagram of this process is shown in Figure 4.

### 4.3. Composite Hierarchical Loss Function Based on Supervised Contrastive Learning and Focal Loss

To fully utilize the hierarchical label information and improve the model’s classification performance on the fine-grained subcategories, this paper proposes a composite loss function, $L_{par-sub}$, as defined in Equation (7). Here, $L_{par}$ represents the parent-category loss, and $L_{sub}$ is the subcategory loss. The core logic of this study is that the ultimate objective is to achieve accurate subcategory classification, while the parent-category labels serve as contextual assistance. Therefore, the subcategory loss implements rigid fine-tuning based on Focal Loss, whereas the parent-category loss adopts a supervised contrastive learning mechanism for a “soft” hierarchical feature constraint. Both objectives are directly summed and optimized synchronously within a single training session, avoiding the introduction of an additional balancing hyperparameter.

(7) $$L_{par-sub}=L_{par}+L_{sub}$$

Specifically, let the dataset be $X=\{x_{1},\ldots ,x_{N}\}$ and the label set be $Y=\{y_{1},\ldots ,y_{N}\}$, where $y_{i}$ is a tuple $\left(y_{i1},y_{i2}\right)$. Here, $y_{i1}$ represents the parent-category label, and $y_{i2}$ represents the subcategory label. As illustrated in the Classification Head in Figure 2, the head consists of two cascaded fully connected layers. The first layer outputs low-dimensional features $f_{1}\in R^{d_{1}}$, and the second layer outputs high-dimensional features $f_{2}\in R^{d_{2}}$. The low-dimensional feature $f_{1}$ is utilized for the feature space computation of the parent-category contrastive loss $L_{par}$, while the high-dimensional feature $f_{2}$ is used for the subcategory classification prediction of the Focal Loss $L_{sub}$.

**Parent-Category Supervised Contrastive Loss:** Since the parent categories in the HOUSED dataset consist of only three classes without an obvious long-tail distribution, employing a traditional rigid cross-entropy loss is suboptimal. Instead, we adapt the InfoNCE Loss function [16] into a supervised contrastive learning framework. In the low-dimensional feature space, samples sharing the same parent category within a batch are treated as mutually positive pairs, while those from different parent categories serve as negative pairs. By minimizing this loss, positive pairs are pulled closer while negative pairs are pushed apart, thereby imposing a “soft” constraint on the parent categories. This process establishes a robust feature representation foundation, akin to pre-training, which significantly accelerates model convergence and facilitates the subsequent subcategory classification. The calculation is defined in Equation (8):

(8) $$L_{par}=-\frac{1}{N}\sum_{i=1}^{N}\log\frac{\sum_{j\in P(i)}\exp\left(\mathrm{sim}\left(f_{i}^{\left(1\right)},f_{j}^{\left(1\right)}\right)/\tau \right)}{\sum_{k\neq i}\exp\left(\mathrm{sim}\left(f_{i}^{\left(1\right)},f_{k}^{\left(1\right)}\right)/\tau \right)}$$

Equation (8) represents the supervised parent-category contrastive loss function designed for our model. Unlike standard self-supervised contrastive learning that relies on a single augmented positive view, our method leverages the true labels to construct multiple positive pairs. Specifically, *N* denotes the total number of samples in the dataset, and *i* is the index of the current anchor sample. The variable $f_{i}^{\left(1\right)}$ represents the low-dimensional feature embedding of the anchor, extracted by the first layer of the classification head. The superscript (1) explicitly denotes its hierarchical origin, distinguishing it from the subsequent layer’s output probability $p_{i}^{\left(2\right)}$.

Furthermore, P(i) denotes the set of indices of all other samples in the dataset that share the same parent category as the anchor, with j∈P(i) indicating the index of a specific positive sample and $f_{j}^{\left(1\right)}$ representing its corresponding feature embedding. The parameter τ is the temperature hyperparameter. In this study, τ is set to 0.07 based on empirical validation. The numerator aggregates the exponential similarities between the anchor and all its positive counterparts. By maximizing this term, the model pulls samples of the same parent category closer in the feature space. Conversely, the denominator aggregates the exponential similarities between the anchor and all other samples *k* in the dataset (where $f_{k}^{\left(1\right)}$ is the feature embedding of sample *k*), generating a repulsive force to push negative samples apart.

Crucially, the condition k≠i is introduced in the denominator to strictly exclude the anchor itself. Because the cosine similarity between an anchor and itself reaches the theoretical maximum of 1, failing to exclude it would result in a massive constant term after exponential scaling (i.e., exp(1/τ)). This “self-similarity” would numerically dominate the denominator, triggering gradient vanishing during backpropagation and preventing the model from effectively learning relative feature differences. Therefore, excluding the anchor is a critical mathematical constraint to ensure the generation of effective push-and-pull gradients.

**Subcategory Focal Loss:** In the fine-grained classification task of subcategories, the traditional cross-entropy loss exhibits a minor penalty distinction between high-confidence and low-confidence prediction regions. Consequently, it struggles to handle confusing and hard-to-classify samples. Furthermore, fine-grained subcategories frequently suffer from severe data imbalance. To address these dual challenges, we adopt the Focal Loss mechanism. By dynamically reducing the relative loss weight of well-classified easy samples, it forces the model to focus its training gradients on hard-to-classify samples (Hard Sample Mining). For our single-label multi-class task, the calculation is simplified and formulated in Equation (9):

(9) $$L_{sub}=-\frac{1}{N}\sum_{i=1}^{N}\omega_{s_{i}}{\left(1-p_{i}^{\left(2\right)}\right)}^{\rho }\log\left(p_{i}^{\left(2\right)}\right)$$

In Equation (9), *N* represents the total number of samples in the dataset, and $s_{i}\in \left\{1,2,\ldots ,K_{sub}\right\}$ denotes the ground-truth subcategory index for the *i*-th sample. To avoid symbol collision with the aforementioned FENNEL algorithm, we employ ω and ρ for the Focal Loss parameters.

The parameter $\omega_{s_{i}}$ is the balancing coefficient for the true subcategory $s_{i}$, specifically designed to mitigate data imbalance. In this study, it is defined based on a normalized inverse class frequency, formulated as $\omega_{s_{i}}=N/\left(K_{sub}\cdot N_{s_{i}}\right)$, where $N_{s_{i}}$ is the total number of training samples in subcategory $s_{i}$. Crucially, the inclusion of the total subcategory count $K_{sub}$ acts as a mathematical normalization factor. It ensures that the expectation of the assigned weights across the dataset remains scale-invariant. This design explicitly amplifies the penalty for misclassifying minority classes without triggering global loss inflation, thereby preventing gradient explosion and preserving learning rate stability.

The parameter ρ (set to 2.0 in this paper) is the focusing parameter that dictates the rate at which easy examples are down-weighted. Finally, this probability $p_{i}^{\left(2\right)}$ is the element value corresponding to the ground-truth category index within the feature vector $f_{i}^{\left(2\right)}$ after the Softmax operation.

## 5. Experiment and Result Analysis

### 5.1. Dataset Description

To validate the robustness of the proposed model and its scalability across different domains, this study utilizes two widely-used hierarchical datasets from the general computer vision field and a constructed hybrid dataset as supplements, in addition to the proposed HOUSED dataset. Detailed descriptions of the datasets considered are provided below:

- **HOUSED:** Contains 30,004 images divided into 9 fine-grained subcategories and 3 parent categories, split into training, validation, and test sets at a 7:2:1 ratio.
- **CIFAR100** [36]**:** Contains 60,000 images with 20 parent categories and 100 subcategories. The data split follows the original paper.
- **tieredImageNet** [37]**:** A subset of ImageNet [38]. To keep the computational cost manageable while preserving a sufficiently complex hierarchical structure, we selected the test split of tieredImageNet as the experimental source pool, which contains 8 parent categories, 160 subcategories, and 206,209 images, and re-split it into training, validation, and test sets at a 7:2:1 ratio.
- **CIFAR100+tieredImageNet:** To verify performance in highly complex scenarios, we mixed 51,000 images from 17 parent categories in CIFAR100 with all the data from the tieredImageNet test set. The resulting hybrid dataset contains 25 parent categories, 245 subcategories, and 257,209 images.

Notably, HOUSED represents the primary application scenario and the domain-specific benchmark of this study, while CIFAR100, tieredImageNet, and the combined CIFAR100+tieredImageNet dataset are introduced to further evaluate the generalizability and scalability of the proposed framework. All four datasets are used in both the ablation experiments and the comparative experiments. The use of the tieredImageNet test split in this study is not intended for direct comparison with prior work using the full tieredImageNet training set, but rather to provide an additional large-scale hierarchical evaluation setting under manageable computational cost. Specifically, the combined CIFAR100+tieredImageNet dataset is approximately 8.6× larger than HOUSED and contains about 27× more subcategories. The consistent improvements across all four datasets further validate the robustness and generalizability of our method beyond the housing domain.

The dataset statistics are summarized in Table 2.

### 5.2. Experimental Baseline Models and Evaluation Metrics

We selected representative baseline models and evaluation settings from CNN-based, Transformer-based, MoE-based, and hierarchical-supervision perspectives to comprehensively evaluate the proposed CS-DisVMoE framework. Specifically, ResNet-50 [39], Inception-v3 [40], and ConvNeXt-Tiny [41] were used as representative CNN-based models. ViT-Tiny [35] was adopted as the Transformer baseline and also served as the backbone for all MoE variants. To evaluate the effectiveness of the proposed routing mechanism, we compared CS-DisVMoE with two representative VMoE architectures: the sparse routing model Expert Choice-Tiny [13] and the soft routing model SoftMoE-Tiny [14]. Using the same ViT-Tiny backbone for all MoE variants allows a controlled comparison of different routing mechanisms under comparable model capacity.

In addition, to evaluate the contribution of hierarchical supervision, we included a flat-classification variant trained only with subcategory Focal Loss and further compared different hierarchical loss configurations. For a comprehensive comparison, we report Top-1 classification accuracy, parameter count, inference FLOPs, convergence behavior, and model depth.

### 5.3. Experimental Setup

All experiments were conducted on four NVIDIA RTX 3090 GPUs. We used Stochastic Gradient Descent (SGD) with a momentum of 0.9, a weight decay of 0.0001, and a batch size of 64 under PyTorch 2.1.0, torchvision 0.16.0, torchaudio 2.1.0, and the CUDA 12.1 build. Transfer learning and mixed precision training strategies were applied. Data augmentation techniques included color jittering, horizontal flipping, and random cropping. Training was executed for 200 epochs to ensure accuracy stabilization. All experiments were repeated five times. Across all datasets, the standard deviations of the repeated runs were within 0.20%, indicating stable experimental results. To avoid overloading the ablation tables, we report the mean and standard deviation only for the main comparative results.

The complete hyperparameter configuration, including model architecture, routing settings, optimization, and hardware, is summarized in Table 3.

### 5.4. Ablation Experiments

**Figure 5:**
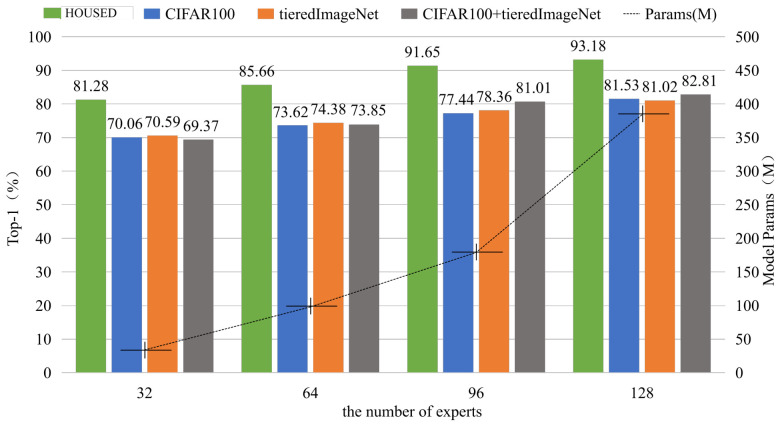
Classification accuracy (bar chart) and model parameter quantity (black line) with different numbers of experts.

First, ablation experiments were conducted on the four datasets to determine the optimal number of experts. As observed in Figure 5, the Top-1 classification accuracy across all datasets consistently improves as the number of experts grows. When the number of experts increases linearly (in steps of 32), the accuracy improves by an average of approximately 3.9% per step. However, the model parameter count also exhibits a steep upward curve. Specifically, scaling from 96 to 128 experts incurs a massive surge in parameters while offering diminishing returns in accuracy gains. Therefore, considering the optimal trade-off between model performance and computational resource efficiency, 96 is selected as the final expert count.

Table 4 presents the ablation study conducted on the proposed innovations. Specifically, ViT-Tiny serves as the baseline non-MoE model and the backbone of our architecture. “+CS-Soft” denotes the integration of the CS-Soft routing mechanism into the baseline. “+FENNEL Expert Distillation” builds upon “+CS-Soft” by incorporating the FENNEL distillation technique. Finally, “+$L_{par-sub}$” represents the further addition of the composite hierarchical loss to the “+FENNEL” configuration.

As observed in Table 4, each proposed innovation contributes to consistent performance gains across both the housing-dimensional urban physical examination and general vision tasks. The improvements are particularly pronounced on the HOUSED dataset and the hybrid dataset (CIFAR100+tieredImageNet). Specifically, on the HOUSED dataset, the CS-Soft routing mechanism yields a 3.08% performance boost, the FENNEL distillation method provides an additional 0.95% gain, and the composite hierarchical loss improves it by a further 1.41%, resulting in a significant cumulative enhancement of 5.44%. Similarly, on the complex hybrid dataset, the three modules contribute sequential gains of 3.27%, 0.77%, and 1.95%, achieving an overall accuracy increase of 5.99%. Across all four evaluated datasets, the proposed method achieves an average cumulative performance improvement of approximately 4.3%. These results thoroughly validate the effectiveness of the proposed model in handling visual classification within complex scenarios.

To determine the default value of γ used in the FENNEL partitioning algorithm, we evaluate its sensitivity across all four datasets. As shown in Table 5, the model maintains stable performance when γ varies from 1.0 to 2.0. The best or near-best results are consistently obtained around γ=1.2–1.5, and γ=1.5 achieves the best performance on HOUSED, tieredImageNet, and CIFAR100+tieredImageNet. Therefore, γ=1.5 is adopted as the default setting in this study. These results indicate that the proposed FENNEL-based expert partitioning is robust to moderate changes in γ.

To further validate the necessity of hierarchical constraints and the design choice of the proposed composite loss, we conduct an ablation study with different loss configurations. As shown in Table 6, using only subcategory Focal Loss removes the parent-category supervision and therefore serves as the baseline without hierarchical constraints. The CE + CE configuration introduces hierarchical supervision by applying cross-entropy losses to both parent and subcategory predictions, while CE + Focal further replaces the subcategory cross-entropy with Focal Loss to address class imbalance and hard samples. Finally, the proposed SupCon + Focal configuration replaces the rigid parent-category cross-entropy with supervised contrastive learning, providing a softer feature-space constraint for parent categories. The results show that adding hierarchical supervision improves performance over Focal-only training, and using Focal Loss for subcategory classification further enhances fine-grained recognition. Moreover, SupCon + Focal achieves the best performance across all datasets, demonstrating that supervised contrastive learning is more effective than standard cross-entropy for parent-category constraint modeling.

In addition to the component-level ablation above, we further validate the necessity of the arctanh transformation in CS-Soft routing by comparing arctanh + softmax with temperature-scaled softmax under different temperatures (T={0.1,0.5,1.0,2.0}). This experiment directly examines whether the proposed non-linear transformation provides benefits beyond simpler temperature-scaled alternatives. As shown in Table 7, under otherwise identical settings, arctanh + softmax achieves the best performance across all datasets without requiring temperature tuning. Note that T=1.0 corresponds to standard softmax, confirming that arctanh provides a meaningful improvement over the baseline.

For subsequent comparative experiments, we evaluate the full **proposed model** (i.e., the network integrating the **CS-DisVMoE modules** and $L_{par-sub}$), and for brevity, we refer to it as the **“Proposed”**.

### 5.5. Comparative Experiment

Table 8 presents a performance comparison across four datasets between the proposed model (incorporating the CS-DisVMoE modules and hierarchical loss) and the existing representative methods introduced in Section 5.2. Observing Table 8, the following conclusions can be drawn:

- The proposed model outperforms the best non-MoE model (ConvNeXt-Tiny) by **3.96%** on the housing-dimensional urban physical examination task, and achieves an average improvement of **1.36%** on the standard general vision datasets (CIFAR100, tieredImageNet). Furthermore, these improvements occur despite a significant difference in model depth, fully demonstrating the advantages of expertization over non-MoE architectures. Although the parameter count increases substantially due to expertization, the incorporation of soft routing combined with expert distillation reduces the inference FLOPs by 1.76G compared to the most lightweight deep non-MoE model (Inception-v3).
- In comparisons with shallow Transformer models based on ViT-Tiny, the proposed method achieves a **5.44%** improvement over the baseline (ViT-Tiny) and a **1.92%** improvement over a competitive MoE model (SoftMoE-Tiny) on the housing-dimensional urban physical examination task. This not only validates the effectiveness of the MoE mechanism in complex scenarios but also supports the effectiveness of our proposed innovations. On general vision tasks, the proposed model achieves an average improvement of **2.88%** over the baseline and **1.29%** over the optimal MoE model, further demonstrating its generalizability.
- The hybrid dataset (CIFAR100+tieredImageNet) increases the recognition difficulty for non-MoE models due to the expanded number of categories and inconsistent sample sizes, resulting in performance lower than that achieved on single-vision datasets. While all MoE models show some level of improvement in recognition capabilities, the proposed method outperforms the best-performing MoE model (SoftMoE-Tiny) by **2.76%**, further indicating its effectiveness in handling complex hybrid vision problems.
- By adopting the FENNEL expert distillation method, under the same base model and expert count settings, the parameter size of the proposed model is reduced by nearly half compared to mainstream existing MoE methods, and the computational FLOPs decrease by 8.33% (because different routing mechanisms are employed, numerous weights in MoE do not participate in actual computations; thus, the reduction in computational FLOPs is less pronounced than the reduction in parameter size).

### 5.6. Training Convergence and Visualization Analysis

**Figure 6:**
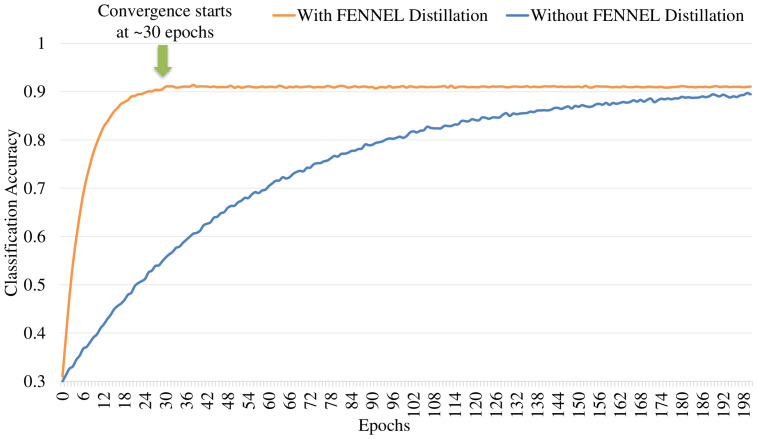
Comparison of training convergence trajectories with and without FENNEL expert distillation.

Figure 6 illustrates the impact of the FENNEL expert distillation method on training convergence. As observed, adopting FENNEL significantly accelerates the optimization process, requiring approximately 170 fewer epochs to achieve comparable classification accuracy than standard random initialization. This efficiency gain primarily stems from mitigating the routing instability typical of early-stage MoE training. Under random initialization, experts lack prior knowledge, leading to inefficient and fluctuating token-to-expert assignments. In contrast, FENNEL distillation initializes the experts with pre-clustered semantic structures transferred from a dense model. By providing these learned feature distributions as a reliable starting point, the model effectively bypasses the “cold-start” phase, enabling the CS-Soft routing mechanism to stabilize rapidly and focus directly on fine-grained task adaptation.

**Figure 7:**
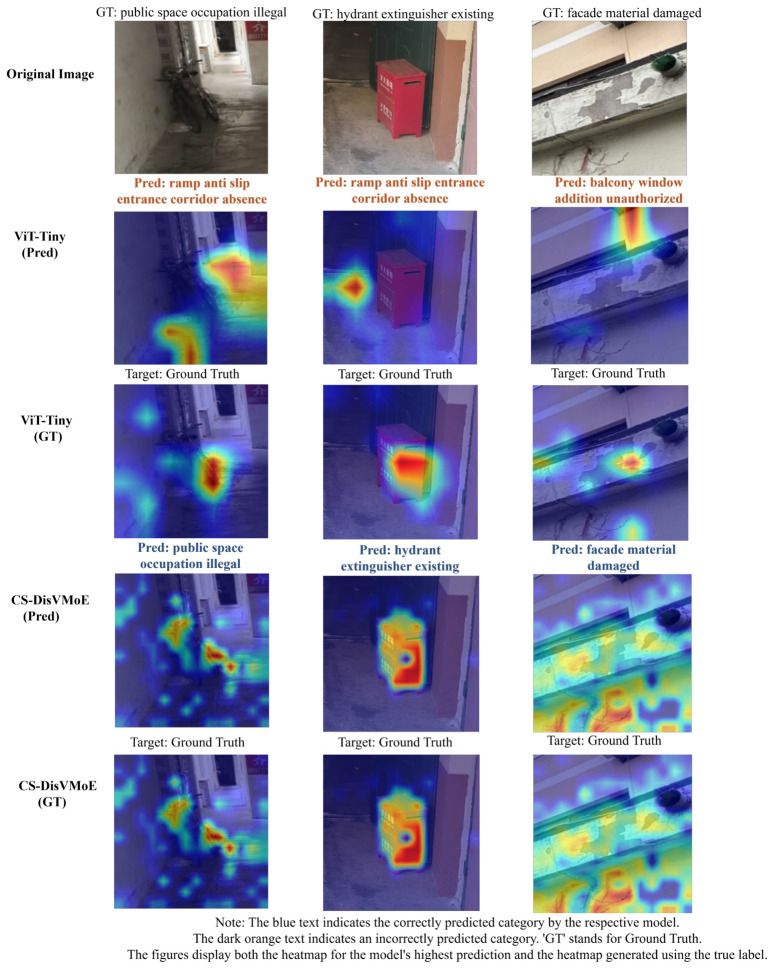
Qualitative comparison of attention maps between the flat-classification variant (**CS-DisVMoE modules** trained solely with the subcategory Focal Loss) and the **full proposed model** incorporating the parent-category supervised InfoNCE loss. The visualization illustrates differences in attention distributions between the two training configurations.

To further illustrate the effect of the proposed hierarchical labeling scheme, Figure 7 compares the attention maps of the baseline flat-classification variant (**CS-DisVMoE module** trained solely with the subcategory Focal Loss) against the **full proposed model**. In computer vision and artificial intelligence studies, attention maps are commonly used as qualitative visualization tools to intuitively observe the feature representation learned by a model, rather than as the primary basis for objective quantitative analysis. Due to the high visual similarities among fine-grained subcategories, the flat variant—lacking the structural constraint of the parent-category supervised contrastive (InfoNCE) loss—tends to exhibit more dispersed attention responses. In contrast, by incorporating the complete $L_{par-sub}$ loss, the full model leverages the parent-category InfoNCE context as a semantic prior, which helps constrain the feature representation within the corresponding parent category. As a result, the attention responses become more concentrated on visually discriminative regions, suggesting that the full model learns more structured feature representations. The quantitative effectiveness of the proposed hierarchical loss is evaluated by the classification ablation results in Table 6, while Figure 7 serves as a qualitative visualization of feature representation differences.

## 6. Conclusions

To advance the automated housing-dimensional urban physical examination, this study introduces the HOUSED dataset and develops a novel hierarchical Vision Mixture of Experts (VMoE) framework. At its core, this framework introduces the **CS-DisVMoE module**, which features a redesigned CS-Soft routing mechanism and employs the FENNEL non-linear streaming graph partitioning method. This core module design effectively accelerates training convergence while significantly reducing parameter overhead.

Furthermore, the overall framework incorporates a composite hierarchical loss function ($L_{par-sub}$) based on Supervised Contrastive Learning and Focal Loss. This integration synchronously accomplishes parent-category feature space alignment and subcategory hard sample mining within a single training session, significantly optimizing the final classification accuracy for fine-grained subcategories. Comprehensive experiments across multiple datasets support the effectiveness and scalability of the proposed framework in addressing complex visual classification tasks.

However, the HOUSED dataset currently contains only about 30,000 samples. Extending to larger-scale urban detection tasks with deeper taxonomies is an important direction for future work. We plan to expand the HOUSED dataset, incorporating additional spatial contexts (e.g., underground facilities, rooftop structures) and more fine-grained defect categories, to further validate the scalability of the proposed framework in complex real-world inspection scenarios.

## Figures and Tables

**Table 1:** Description of parent categories and subcategory labels with their respective sample sizes and semantic meanings.

Parent Category	Readable Class Name	Original Dataset Label (Size)	Semantic Description
passage_way (17,491)	Public Corridor Blocked	public_space_occupation_illegal (4997)	The public corridor is physically blocked by unauthorized objects.
	Missing Anti-slip Ramp	ramp_anti_slip_entrance_corridor_absence (9274)	The entrance lacks age-appropriate accessibility modifications.
	Damaged Staircase	step_missing_damaged (1606)	The staircase exhibits visible structural damage.
	Damaged Floor Surface	roof_floor_finish_damaged (1614)	The floor surface has potholes and uneven cement.
interior_wall (5226)	Fire Extinguisher Present	hydrant_extinguisher_existing (1237)	Visible fire protection facilities are present.
	Damaged Wall Plaster	facade_material_damaged (3487)	The wall plaster is peeling with visible cement detachment.
	Missing/Damaged Extinguisher	hydrant_extinguisher_missing_damaged (502)	The fire hydrant equipment is incomplete or damaged.
external_structure (7287)	Exterior Elevator Addition	elevator_installation_absence_addition (778)	An exterior elevator has been newly installed.
	Unauthorized Bay Window	balcony_window_addition_unauthorized (6509)	Unauthorized bay windows or security cages are added.

**Table 2:** Hierarchical label structure of the datasets, including the number of parent categories, subcategories, and total samples.

Dataset	No. of Parent Categories	No. of Subcategories	Number of Samples
HOUSED	3	9	30,004
CIFAR100	20	100	60,000
tieredImageNet	8	160	206,209
CIFAR100+tieredImageNet	25	245	257,209

**Table 3:** Complete hyperparameter configuration.

Hyperparameter	Value
Backbone	ViT-Tiny [35], 12-layer Transformer
Embedding dimension *d*	192
MLP hidden dimension	768
Input resolution	224 × 224, patch 16 × 16, 196 patch tokens + 1 class token
Dense layers	8 (first 8 layers retain original MLP)
MoE layers	4 (last 4 layers replaced with CS-DisVMoE)
Number of experts *n*	96 (determined by the ablation study )
Slots per expert *p*	1 (following Soft MoE [14])
Total slots n×p	96
Routing dimension	192 (matches the ViT-Tiny embedding dimension)
$W_{Q},W_{K}$	[192,192] (learnable projection matrices)
FENNEL γ	1.5 (selected based on sensitivity analysis)
τ	0.07 (InfoNCE contrastive loss temperature)
ρ	2.0 (Focal Loss focusing parameter)
Optimizer	SGD, momentum = 0.9, weight decay = 0.0001
Batch size	64
Training epochs	200
Data augmentation	Color jittering, horizontal flip, random crop
Hardware	4× NVIDIA RTX 3090

**Table 4:** The ablation experiments of the three innovation points proposed in this paper (%).

Dataset	ViT-Tiny (Base)	+CS-Soft	+FENNEL Expert Distillation (CS-DisVMoE)	+$L_{\mathrm{par}-\mathrm{sub}}$ (SupCon + Focal Loss)
HOUSED	86.21	89.29 (+3.08)	90.24 (+0.95)	91.65 (+1.41)
CIFAR100	74.92	76.32 (+1.40)	76.89 (+0.57)	77.44 (+0.55)
tieredImageNet	75.12	77.65 (+2.53)	77.94 (+0.29)	78.36 (+0.42)
CIFAR100+tieredImageNet	75.02	78.29 (+3.27)	79.06 (+0.77)	81.01 (+1.95)

**Table 5:** Sensitivity analysis of the FENNEL parameter γ across different datasets. Top-1 accuracy (%) is reported; bold values indicate the best performance for each dataset, and “Default” marks the adopted setting.

Dataset	γ=1.0	γ=1.2	γ=1.5 Default	γ=1.8	γ=2.0
HOUSED	91.28	91.52	**91.65**	91.55	91.35
CIFAR100	77.38	**77.55**	77.44	77.52	77.32
tieredImageNet	78.15	78.30	**78.36**	78.28	78.10
CIFAR100+tieredImageNet	80.82	80.95	**81.01**	80.92	80.75

**Table 6:** Ablation study on hierarchical constraints and parent-category loss design. Top-1 accuracy (%) is reported. CE denotes cross-entropy, and SupCon denotes supervised contrastive learning.

Configuration	HOUSED	CIFAR100	tieredImageNet	CIFAR100+ TieredImageNet
Focal only	89.82	75.85	76.92	78.54
CE + CE	90.24	76.31	77.23	79.05
CE + Focal	90.71	76.88	77.61	79.62
SupCon + Focal	**91.65**	**77.44**	**78.36**	**81.01**

**Table 7:** Ablation comparison of arctanh versus temperature-scaled softmax in CS-Soft routing. Top-1 accuracy (%) is reported.

Configuration	HOUSED	CIFAR100	tieredImageNet	CIFAR100+ TieredImageNet
Softmax, T=0.1	89.82	75.95	76.85	78.92
Softmax, T=0.5	90.68	76.85	77.52	79.78
Softmax, T=1.0	90.92	77.15	77.88	80.15
Softmax, T=2.0	90.45	76.68	77.35	79.52
arctanh + softmax	**91.65**	**77.44**	**78.36**	**81.01**

**Table 8:** The comparative experiments of the proposed model with various existing representative models. Top-1 accuracy is reported as mean ± std over five runs.

Dataset/Metric	Deep Models (CNN)			Shallow Models (ViT)			Proposed
	ResNet-50	Inception-v3	ConvNeXt-T	ViT-Tiny	Expert Choice	SoftMoE-T	CS-DisVMoE+$L_{\mathrm{par}-\mathrm{sub}}$
**Accuracy (%, mean ± std)**							
HOUSED	86.43 ± 0.12	87.59 ± 0.10	87.69 ± 0.09	86.21 ± 0.13	88.62 ± 0.11	89.73 ± 0.10	**91.65 ± 0.07**
CIFAR100	75.39 ± 0.11	75.87 ± 0.10	76.36 ± 0.09	74.92 ± 0.12	75.81 ± 0.10	75.91 ± 0.09	**77.44 ± 0.06**
tieredImageNet	75.71 ± 0.16	76.36 ± 0.14	76.73 ± 0.13	75.12 ± 0.17	76.88 ± 0.14	77.32 ± 0.12	**78.36 ± 0.09**
CIFAR100+tiered	75.56 ± 0.18	76.14 ± 0.16	76.33 ± 0.15	75.02 ± 0.18	76.97 ± 0.15	78.25 ± 0.13	**81.01 ± 0.10**
**Model Properties**							
Model Depth	50	42	23	12	12	12	12
Params (M)	26	24	29	6	354	354	179
FLOPs (G)	4.13	2.86	4.46	1.10	1.20	1.20	1.10

## Data Availability

HOUSED: Housing-dimensiOnal visUal inSpection imagE Dataset. V2. Science Data Bank. https://doi.org/10.57760/sciencedb.28941 (accessed on 26 May 2026).
